# Is ovarian cancer surgery stuck in the dark ages?: a commentary piece reviewing surgical technologies

**DOI:** 10.1038/s41416-020-01035-9

**Published:** 2020-08-24

**Authors:** David L. Phelps, Srdjan Saso, Sadaf Ghaem-Maghami

**Affiliations:** 1 Imperial College Healthcare NHS Trust, London, W12 0HS UK; 2 Imperial College London, London, W12 0NN UK

**Keywords:** Ovarian cancer, Surgical oncology

## Abstract

Ovarian cancer surgery endeavours to remove all visible tumour deposits, and surgical technologies could potentially facilitate this aim. However, there appear to be barriers around the adoption of new technologies, and we hope this article provokes discussion within the specialty to encourage a forward-thinking approach to new-age surgical gynaecological oncology.

## Main

Epithelial ovarian cancer (EOC) survival is improved with minimisation of disease at surgery, especially for women achieving complete surgical cytoreduction (CSC). Radicality per se is, however, not always the answer to better survival and may increase morbidity. For example, it is now clear that radical lymphadenectomy in OC surgery is not necessary when there is no palpable lymphadenopathy.^1^ Neoadjuvant chemotherapy followed by surgery results in less radical surgery and less surgical morbidity. The Trial of Radical Upfront Surgical Therapy will answer the outstanding questions regarding neoadjuvant chemotherapy versus primary cytoreductive surgery in the radical surgical setting.^2^ The results of these trials will inevitably leave us seeking better ways to achieve CSC, to minimise surgical morbidity, to identify involved lymph nodes and to offer a more personalised surgical approach. New technologies have been widely accepted into other surgical specialities, such as neuronavigational systems in neurosurgery, robotics for prostatectomies and capsule endoscopy in gastroenterology, but less so for EOC. In this modern era of technology and artificial intelligence, there are a multitude of surgical technologies emerging for EOC. This commentary piece discusses current and emerging technologies, which may improve CSC rates, improve intra-operative diagnostics and offer surgical advantages over current methods.

### Mass spectrometry (MS) technologies

Intra-operative diagnosis of the malignant potential of an early-stage ovarian mass is difficult, but more obvious in metastatic disease. For younger women, with low-stage disease, it is essential that accurate diagnosis can be achieved before clearance of all reproductive organs. New intra-operative tumour diagnostic technologies are emerging and are based on MS systems.

MS-based systems rely on tissue lipidomes as ‘fingerprints’ that can report histological diagnosis in a few seconds. Desorption electrospray ionisation MS (DESI-MS) sprays solvent onto tissue and analyses secondary solvent ions that rebound from the tissue.^3^ This technology has excellent diagnostic accuracy for EOC ex vivo with 99.6% predictive accuracy on a pixel-by-pixel basis.^3^ The MassSpec Pen incorporates DESI-MS and yields impressive diagnostic accuracy in the laboratory (94.7% diagnostic accuracy, area under the curve 0.98 [normal ovary versus EOC]).^4^ Rapid evaporative ionisation MS utilises existing electrosurgical diathermy, as the surgical intelligent knife (iKnife), to create tissue aerosols rich in lipids during dissection.^5^ The iKnife has been used for EOC diagnostics both ex vivo and in vivo with impressive accuracy (97.4% sensitivity, 100% specificity [normal ovary versus EOC]).^5^ Further validation lead to excellent EOC diagnostic accuracy (100%). Furthermore, borderline ovarian tumours were distinguishable from EOC (sensitivity 90.5%, specificity 89.7%),^5^ which has important implications for women wishing to preserve fertility. Laser-based technologies, which sample tissue only a few microns across, are in development and could lead the way towards a new surgical era of achieving complete *microscopic* cytoreduction. One major benefit of these technologies is their ability to detect tumour tissue at the microscopic level.^3–5^ It is widely accepted that clearance of *macroscopic* disease portends improved survival— perhaps microscopic clearance of disease should be the next paradigm shift in cytoreductive surgery? Currently, it is unclear which system(s) will be adopted, as none of the devices are commercially available or approved for use outside of a research setting as yet.

### Robotic systems

Robotic systems improve operating ergonomics, allowing articulation of instruments beyond the normal constraints of a wrist. Since the first robot in 1985 there has been an explosion in the market for robots, but robot-assisted surgery in EOC has not been widely integrated into surgical practice.

One meta-analysis, comparing laparotomy and laparoscopy robotic approach for EOC surgery, found no significant difference in outcomes.^6^ While FIGO (International Federation of Gynaecologist and Obstetrics) stages I to IV were included in a total of eight studies, only four studies included any survival data. The study failed to show oncological safety and recurrence by pathological stage or histologic types.^6^ There was, however, a reduction in blood loss, post-operative hospital stay and complications, but these benefits were only valid when comparing robotic surgery to laparotomy—there was no advantage to robotic surgery over laparoscopy.^6^ Another study compared the effectiveness of robot-assisted laparoscopy and traditional laparoscopy in stage I EOC and found there to be a significantly lower rate of conversion to open surgery (7.2% versus 17.9%, *P* < 0.001; adjusted odds ratio [OR]: 0.49, 95% confidence interval [CI]: 0.33–0.73).^7^ After multi-variable adjustments, the study failed to show any meaningful differences between survival when comparing robotic surgery with traditional laparoscopy.^7^

The robot may be useful in the neoadjuvant setting. Robot-assisted surgery achieved optimal cytoreduction rates of 100% (≤1 cm residual disease) with 82.5% having no residual disease (*n* = 57). Post-operative recovery was rapid (84% discharged home within two days of surgery) and there was improvement in overall survival (OS) and progression-free survival (PFS) (robot versus laparotomy; OS: 37.8 versus 47.2 months, *P* = 0.04/PFS: 13.9 versus 20.6 months, *P* = 0.005). The authors concluded that in very select patients the use of robotic interval cytoreduction is feasible.^8^ However, this trial was significantly limited by its small numbers, retrospective data collection and non-randomised design.

The role for robotic surgery differs between the varying stages of EOC and in the primary or neoadjuvant surgical setting. Large prospective randomised controlled trials assessing robots in select cohorts of women need to be established before firm conclusions about potential benefits of robots in EOC surgery can be reached.

### Fluorescent imaging systems

Indocyanine green has been used to reliably identify lymphatic drainage in early ovarian cancers, including sentinel lymph node (SLN) identification. One study identified SLNs in 88.9% of primary EOC surgeries and in four patients with positive nodes 100% had the positive SLN identified.^9^ The trial of lymphadenectomy in patients with advanced ovarian neoplasms (LION) showed no survival benefit in systematic lymphadenectomy in macroscopically normal lymph nodes, for women achieving CSC.^1^ There was only survival benefit when removing bulky nodes. This raises the possibility that women who have small involved lymph nodes will miss out on the potential survival benefit of involved lymph node excision if involved nodes cannot be identified. Sampling of SLNs would therefore be a logical next step to establish prognostic effect of removing involved SLNs. It is feasible to specifically excise SLNs and perform targeted compartmental lymphadenectomy to reduce morbidity.^10^

Other intra-operative fluorescent imaging technologies to identify tumour deposits have been used in EOC surgery with relative success. Folate receptor-α, expressed by 90–95% of EOCs, can be successfully detected intra-operatively with near-infrared technology.^11^ One study showed that an additional 29% of malignant lesions were resected using fluorescent imaging.^11^ Furthermore, a nanomolecular probe coupled to a bacteriophage, which binds to the SPARC protein, allows intra-operative imagery to guide debulking.^12^ This associated with improved survival in murine models (control versus image-guided, 18 days versus 40.5 days survival; *P* = 0.039, hazard ratio: 0.26, 95% CI: 0.07–0.93).^12^

Operating post chemotherapy, when tumour has undergone calcification and fibrosis, makes tumour identification more challenging. This is an important distinction to make during surgery, as achieving CSC is always the aim. This distinction is especially important since the publication of DESKTOP III data, which shows improved OS and PFS in women achieving CSC in the recurrent setting post chemotherapy.^13^ The fluorescent imaging systems have not yet been trialled in the recurrent setting—this would be an interesting avenue of investigation.

### PlasmaJet™ technology

PlasmaJet utilises kinetic energy and highly controlled thermal effects to vaporise microlayers of tissue.^14^ The high-energy jet of argon plasma dissects without applying an electrical current to the tissue, limiting thermal spread and adjacent tissue damage.^14,15^ This controlled depth of dissection works well on the surface of the bowel, peritoneum and diaphragmatic surfaces. Reviews of PlasmaJet’s use in EOC report no harm or additional complications with CSC achieved in 79.0–84.3% of patients.^15,16^ Bowel fistulae were not observed when using PlasmaJet on intestinal mesentery or bowel serosa. The authors conclude that the device is well suited for peritoneal stripping and treating diaphragmatic disease (resection or ablation), in addition to bowel serosa and mesentery stripping.^15,16^

Carcinosis on the bowel mesentery, serosa and diaphragm are among the most common sites that surgeons are unable to treat; thus, it could be assumed that PlasmaJet would increase CSC rates. However, surgical expertise is required to operate on these sites and the introduction of a new device alone would not necessarily alter CSC rates. Training of surgeons and a shift in surgical philosophy would need to be achieved to have a significant impact. A randomised controlled trial was registered in 2013 to evaluate the utility and efficacy of PlasmaJet in achieving CSC, but recruitment ended in 2017 and the results are overdue.^17^ Nevertheless, the published data thus far from other studies appear to suggest that PlasmaJet is safe and it would be worthwhile testing it further in appropriately controlled trials to evaluate its potential for EOC surgery.

### Conclusion

There are many exciting new and emerging technologies, some of which have been shown as effective in EOC surgery. Very few (if any) of these concepts have been widely accepted into surgical practice by gynae-oncologists, perhaps due in part to commercial availability, but maybe also a reluctance to adopt new technologies. It is essential that, as gynae-oncology surgeons, we stay abreast of these rapidly evolving technologies in this modern surgical era to ensure that we are offering the best care to our patients. As a community we can engender a forward-thinking approach by showing a willingness to participate and collaborate in multi-centre clinical trials that test these new ideas. It is clear that surgery in the modern age is rapidly changing and we should embrace this evolving world and the potential benefits that may come with it.

## Data Availability

Not applicable.
